# What Community Members With Chronic Illness Teach Future Healthcare Professionals in a Longitudinal Interprofessional Education Program: A Focus Group Study

**DOI:** 10.1111/tct.70181

**Published:** 2025-08-15

**Authors:** Rachel White, Maria Brucato, Amber King, Anne B. Mitchell, Nethra S. Ankam

**Affiliations:** ^1^ Sidney Kimmel Medical College Thomas Jefferson University Philadelphia Pennsylvania USA; ^2^ Jefferson Center for Interprofessional Practice & Education Philadelphia Pennsylvania USA; ^3^ Jefferson College of Pharmacy Thomas Jefferson University Philadelphia Pennsylvania USA; ^4^ Jefferson College of Nursing Thomas Jefferson University Philadelphia Pennsylvania USA

## Abstract

**Introduction:**

Nearly 50% of Americans have at least one chronic illness. Preparing future healthcare providers for interprofessional collaborative practice (IPCP) and person‐centred care through community‐based interprofessional education (IPE) can improve outcomes for this growing population. Partial programme theories predict that patients' participation as teachers in health professions education contributes to students' development of patient‐centredness. Role theory asserts that behaviours are shaped by assigned roles; therefore, community members with chronic illness who assume roles of health mentors in an IPE curriculum are predicted to teach students about their patient experiences. Yet, this teaching outcome and the lessons health mentors teach in semi‐structured student‐led meetings are underexplored.

**Goals:**

This project explored what community members with chronic conditions teach future healthcare professionals about their conditions/disabilities and healthcare access and quality in a health mentors IPE programme.

**Methods:**

Thirty‐eight health mentors participated in focus groups and were surveyed for demographic information. Directed content analysis was performed, drawing on prior research, programme theory and core competencies in IPCP and disability.

**Results:**

Four themes were identified related to barriers in accessing healthcare: environmental factors, insurance, cost and time. Themes regarding chronic conditions, disabilities or healthcare quality included education about the patient journey, communication, person‐centred care, team collaboration, advocacy, empathy, impact of providers' words and shared decision‐making.

**Conclusion:**

Partial programme theories suggest these lessons are especially impactful when delivered by patients. Aligned with role theory, our findings suggest that clinical teachers can rely on health mentors to share key lessons with students within a semistructured student‐led context.

## 1 Introduction

Over 50% of the American population, 129 million people, have at least one chronic illness; this number is projected to increase as the population ages and people live longer [1, 2, 3]. Patients encounter a variety of barriers to care for their chronic conditions, including financial constraints and insurance challenges, time burdens for logistical aspects of care, disagreements or contradictory information about care plans among healthcare team members and providers overlooking or ignoring their concerns [4]. Various strategies for enhancing the healthcare quality and outcomes of people with chronic conditions are still being investigated, and care coordination and self‐management interventions delivered by interprofessional healthcare teams may improve chronically ill patients' care experiences and health behaviours [5]. Patients with chronic illness report profoundly positive experiences with interprofessional collaborative practice models, describing benefits like access to a collaborative network of healthcare professionals with a diversity of expertise, enhanced feelings of trust for and respect by professionals on healthcare teams, holistic care and active participation in care planning [6]. Thus, interprofessional education (IPE) offers a promising approach for preparing future healthcare professionals to provide interprofessional team‐based person‐centred care to improve outcomes for a rapidly growing number of chronically ill patients.

One IPE model that may be particularly serviceable in these efforts is longitudinal community‐based IPE, which pairs interprofessional student teams with community volunteers (health mentors) living with chronic illness and/or disability (e.g., [7, 8, 9]). Reported impacts of these types of IPE programmes on health professions students include improved attitudes toward interprofessional teamwork (e.g., [10, 11, 12, 13]), increased positive perceptions of healthcare team members from different professions [14] and the opportunity to practice and demonstrate teamwork skills (e.g., [15, 16]). According to partial programme theories of de Groot et al. [17], longitudinal community‐based IPE programmes also provide educational conditions for increased person‐centredness in learners: The theories predict that (1) when patients take the role of an *informant* and share about their condition(s) in daily life, students are able to imagine patients' lives more completely; and (2) when patients take the role of a *teacher*, and the student takes on the role of a *chronicler*, students demonstrate changes in what knowledge is considered valuable, integrating various perspectives aside from their own and developing narratives with patients [17].

Specifically, de Groot and colleagues draw on role theory to describe the conditions in which longitudinal community‐based IPE programmes should result in successful learner development of person‐centred care. According to role theory, people's behaviours are shaped by the social roles that they occupy within a specific context (e.g., [18]). Therefore, for a patient to exhibit behaviours associated with the role of the *teacher* or *informant*, it may be particularly important for IPE programmes to orient all participants (students, faculty and patients) to view health mentors as serving in that role, rather than that of the patient. While institutions each follow specific recruitment and training strategies for health mentors relevant to their context, these processes typically advertise the experience as an opportunity for community members to teach future healthcare professionals about the importance of skills such as teamwork, collaboration and person‐centred care [7, 19]. This introduces the role of teacher early in the recruitment process.

Indeed, community volunteers report that they value teaching future healthcare professionals about person‐centred care, their experiences living with chronic illness and their expectations of providers through storytelling and knowledge sharing [9, 20, 21]. However, little is known about precisely what types of experiences health mentors share with their student teams regarding barriers and facilitators to receiving care for their chronic conditions, and if they are representative of those reported in the clinical literature. Health mentors' IPE programmes can involve hundreds of students and health mentors, making it logistically challenging to provide structured guidance for formal instruction on multiple lesson areas to community volunteers [7]. However, many IPE curricula directors acknowledge patients as experts in their own health and life and therefore consider them well‐prepared to serve in the role of teachers [19]. Aligned with role theory, empowering people with chronic illness to take on the role of teacher may be enough to elicit key lessons, even without health educators providing structured lesson plans.

*Empowering people with chronic illness to take on the role of teacher may be enough to elicit key lessons*.

Thus, there are two gaps in the existing literature this study sought to address. First is an evidence gap regarding whether current training practices and orientation to roles in a well‐established health mentors program result in expected teaching outcomes; second, is a knowledge gap regarding what types of lessons health mentors teach students in meetings, as reported from their perspective. Cataloguing the experiences health mentors share with students in these areas can shed light on whether or not greater guidance to prepare health mentors for the role of teacher is necessary to strengthen IPE curricula and facilitate positive change in care for chronically ill populations. It may also further our understanding of what barriers and facilitators healthcare chronically ill patients face when interfacing with healthcare systems. Our interprofessional research team conducted focus groups with health mentors to explore their experiences in a long‐standing health mentors' IPE programme in the United States. The specific aim of the present study was to analyse two sections of the focus group data to investigate what health mentors teach healthcare professionals in training about living with or caring for someone with a chronic illness, including barriers and facilitators they have experienced when seeking healthcare.

## 2 Methods

This study followed recommended guidelines for qualitative interview studies to determine a sufficient sample size based on the informational power of the sample [22] and followed the Consolidated Criteria for Reporting Qualitative Research [23]. Focus groups have several methodological strengths compared to an interview approach in general and for the present study in particular, which motivated our use of this method. First, according to a health mentor in an advisory role and information from staff and faculty who worked closely with the population of health mentors sampled in the present study, research occurring in a social group setting was preferable to health mentors and therefore provided a mutually beneficial and positive experience for research participants. Second, group interactions during focus groups encourage idea generation and discussion, which is advantageous for capturing a breadth of perspectives.

### 2.1 Research and Educational Context

This study was conducted at a private research university and academic health centre in an urban setting in the United States and recruited study participants who were involved in the university's Health Mentors Program (HMP; [10, 19]). HMP is a 1.5‐year IPE course that consists of three modules relating to the impact that individual, interpersonal, environmental and societal factors have on the well‐being of those with chronic illness. The course enrolls over 1500 students from 12 professions, including student athletic trainers, counseling and behavioural health professionals, dietitian nutritionists, genetic counselors, medical laboratory scientists, nurses, occupational therapists, pharmacists, physical therapists, physicians, physician assistants and speech‐language pathologists. Students are placed in small interprofessional groups of 5–7 and paired with a health mentor, a volunteer from the community who is living with and/or caring for someone with one or more chronic illnesses or disabilities.

Health mentors included in the present study met definitions for Towle et al.'s [8] spectrum of involvement at Level 3 (shares their experience with students) and Level 4 (is given preparation for specific teaching role, may question students during meetings and are invited to provide feedback and evaluations of students' teamwork). The curriculum also emphasized to students the principle that patients in general are teachers, being the experts in their own health and life [19]. Health mentors have the option to complete their introductory meeting with their student team in person and are reimbursed for this travel; otherwise, the volunteer role does not have additional expenses for the health mentor, nor do health mentors receive additional compensation. The learning goals for students in HMP are to understand how an individual's complex social environment interacts with their well‐being, recognize the importance of person‐centred care, collaborate with students from other healthcare professions and gain an understanding of and respect for the roles of various healthcare professionals. Prior studies have found that students meet these learning objectives (e.g., [10, 11]).

In preparation to meet with students, health mentors complete an introductory meeting with a programme coordinator, who introduces the goals of the programme, roles and responsibilities of the health mentor and students, describes the meetings with the students and the assignments to be completed by the students in collaboration with the health mentor. Health mentors are encouraged to share their health care journey, discuss how they live and manage their chronic conditions and disabilities and discuss how they stay well. Mentors are invited to provide programme feedback informally at any point during the programme and formally at specific points during the programme; feedback may be related to the programme in general or about specific students or teams. Mentors are empowered to provide direct feedback to student teams if desired; they are invited to complete a standardized team assessment after 1 year of working with students.

Meetings with health mentors are student‐led. Students are provided with information to guide the interaction, including a brief biography of their health mentor, the goal of the interaction and conversational prompts to help guide the meeting. Students are asked to find out about the health mentor's life, relationships, community and health beliefs. In their second meeting, they are asked to complete an environmental assessment of the mentor's home, and explore further what matters to the mentor in their life. Faculty debrief with teams in groups of 4–5 teams after all teams have completed their meetings.

### 2.2 Participant Characteristics

The purposive sample included a total of 38 participants (see Table 1) who were health mentors for a median of 4 years (Mean = 4.76, SD = 3.23, Min. = 0, Max. = 12) and had a median of 1 chronic condition (Mean = 2.11, SD = 1.78, Min. = 0, Max. = 7). Nearly half (47%) of participants were diagnosed with at least one chronic condition 10 or more years before joining HMP, and over a third had been diagnosed between 1 and 5 years prior to joining the programme (see Table 2). When asked if they share their experiences as a patient, caregiver or both during meetings with students, most participants in the sample reported sharing from primarily the patient perspective (66%), about a quarter reported sharing their experiences both as a patient and caregiver (24%) and one participant reported that they share exclusively from the caregiver perspective (3%). Participants were referred to HMP through a variety of mechanisms (see Table 2), including through their healthcare provider or another healthcare professional (42%), from programme recruitment efforts (24%) and from current health mentors (13%).

**TABLE 1:** Participant demographics.

Demographic	*N*	Percent
***Age range***		
25–34	1	2.6%
35–44	2	5.3%
45–54	5	13.2%
55–64	5	13.2%
65–74	11	28.9%
75–84	11	28.9%
No response	3	7.9%
***Sex***		
Female	15	39.5%
Male	20	52.6%
No response	3	7.9%
***Race and/or ethnicity*** ^**a**^		
African American/Black American	7	18.4%
Asian or Pacific Islander	1	2.6%
Caucasian/White	28	73.7%
Hispanic/Latino	0	0%
Indian or Alaskan Native	0	0%
Multiracial	1	2.6%
No response	3	7.9%
***Identify as having a disability***		
Yes	15	39.5%
No	17	44.7%
Unsure	2	5.3%
No response	3	7.9%

*Note:* Categories are presented in alphabetical order. Percentages calculated out of *N* = 38.

^a^ Percentages do not total 100 as participants could select multiple options from this category.

**TABLE 2:** Participant involvement with the Health Mentors Program (HMP).

Survey question	*N*	Percent
***Method of referral to the HMP***		
From my provider/another healthcare professional	16	42.1%
From a recruitment session/programme faculty or staff	7	18.4%
No response/could not remember	6	15.8%
From a current health mentor	5	13.1%
From a posted flyer/internet	2	5.3%
From a friend/neighbour	2	5.3%
***Volunteer in HMP to share experience as***		
A patient	25	65.8%
A caregiver	1	2.6%
Both a patient and caregiver	9	23.7%
No response	3	7.9%
***Years diagnosed with at least one chronic condition before HMP***		
Less than 1 year	2	5.3%
1–5 years	12	31.6%
6–10 years	4	10.5%
10+ years	16	42.1%
No response	4	10.5%

*Note:* Percentages calculated out of *N* = 38.

Abbreviation: HMP = Health Mentors Program.

### 2.3 Recruitment

In June of 2024, all 133 health mentors who were working with student teams that completed their first academic year of HMP were contacted via mailed or online survey to ask about their interest in continuing with those student teams for a third and final semester. In the survey, health mentors were also provided with a checkbox to express interest in learning about the present study. Fifty‐three (40%) requested more information and were contacted via phone and/or email according to their preference and provided study details and scheduling information. Of the 53 contacted, 38 health mentors (72%) were scheduled for a focus group session. The rate of attrition from scheduling to the study session date was 5% (*n* = 2). Additionally, two health mentors brought family members with them to the study session who were not recruited by the study team but were interested in participating. One was also a seasoned health mentor, and one was starting as a first‐time health mentor in the upcoming academic year; therefore, both met study inclusion criteria and were retained in the sample.

The sample of 38 health mentors who participated in the present study did not differ more than would be expected by chance from the 95 health mentors who did not participate on variables of gender, years served as a volunteer in the programme or number of chronic conditions (*p*s > 0.05). We did not have data from nonparticipants regarding their identification as having or not having a disability for comparison.

### 2.4 Informational Power

Consistent with guidelines described by Malterud et al. [22], our purposive sample provided a high level of informational power based on the study's position along five dimensions: (1) Broadness of study aim was moderate, investigating a breadth of experiences from a narrow population and context; (2) Specificity of knowledge, experiences and characteristics, among participants was balanced, including variety in years of experience with HMP, years managing chronic conditions and demographic characteristics; (3) Theory and prior research were applied in study planning and the analysis approach; (4) Quality of focus group dialogue was high, facilitated by researchers with high familiarity of the programme and population as well as training in semistructured interview techniques; and (5) Analyses were conducted across multiple cases via directed content analysis.

### 2.5 Procedure

Five single timepoint 90‐min semistructured focus group sessions of five to nine participants each were held in August 2024; two were conducted in person in a quiet classroom on the university campus with refreshments and travel reimbursement for participants, and three were held virtually via videoconference. Health mentors were assigned to focus group sessions based on their preferred format (in‐person vs. virtual) and their availability. Prior to the focus group session, each participant was asked to complete a demographics questionnaire (see Appendix S1) and provided their verbal informed consent for study participation.

The semistructured focus group guide consisted of four main sections asking health mentors to describe (1) the events that led them to become a health mentor; (2) their perceptions of HMP and their role in the programme; (3) experiences they share with students about (3a) having and/or caring for someone with chronic illness or disability, (3b) barriers and supports to receiving the healthcare they need and (3c) qualities of effective and ineffective healthcare teams; and (4) their observations pertaining to student team growth over the course of the HMP (see Appendix S2). It was drafted by study team members (M.B. and R.W.) and reviewed and revised by additional study team members (A.B.M. and N.S.A.) and one health mentor prior to its use in the present study. Each focus group was led by two co‐moderators (R.W., a female medical student; and M.B., a female research professional experienced in focus group interviews) who both had a robust understanding of HMP's learning objectives and structure. The co‐moderators did not have a previously established relationship with any study participants before study recruitment. Focus group sessions were audio recorded, transcribed using Microsoft Word's auto‐transcription feature and reviewed and updated for accuracy by the study team before data analysis.

For the purposes of the present investigation, sections 3a and 3b were analysed to explore what experiences health mentors teach their students about the patient/caregiver experience, and specifically, if they choose to share with students any barriers or facilitators to care that they have experienced when managing a chronic condition.

### 2.6 Data Analysis

Transcript data from sections 3a and 3b of the focus group (see Appendix S2) were analysed using qualitative directed content analysis [24]. Two independent raters coded transcript data; inter‐rater reliability was calculated, and coding review meetings were held regularly to discuss and resolve all discrepancies through consensus. Data were coded in Microsoft Excel. The unit of analysis for qualitative coding was defined as a complete thought. This could encompass a single sentence or multiple sentences that collectively represent a consistent idea or concept. Each chunk of text was coded based on its thematic coherence, ensuring that each unit captured a distinct and meaningful segment of the data.

An initial codebook was developed a priori based on prior theory [17] and research (i.e., [4, 6]), as well as competencies for interprofessional collaborative practice [25] and disability clinical care [26]. Throughout the coding process, the codebook was updated twice based on new themes identified by the research team during coding review meetings. These included the addition of an ‘insurance’ code, differentiated from an existing ‘costs’ code and the addition of an ‘impact of provider's words on patients’ code (see Appendix S3). Any code that coders agreed was represented by at least five thought units in the focus group data was considered as a theme.

## 3 Results

Across five focus group transcripts, the research team identified 339 unique thoughts, and 186 were coded as ‘other’. All thoughts coded as ‘other’ were discussed during coding review meetings, and relevance to the focus of the study was determined by consensus. Thought units coded as ‘other’ included those without full semantic structure including ‘uhs’ or ‘ums’, artefacts of the social setting of the focus group (e.g., ‘One of the biggest things—Oh sorry, go ahead’, ‘And then I lost my second thought’ and ‘Can you hear me?’), and a small number of other comments that did not clearly meet a definition in our codebook (e.g., ‘Yeah, to me, my wife doesn't think I'm very livable today’, ‘The sicknesses that I have, doctor [name] said they're all DNA related’ and ‘My wife … was also involved when she was alive. And we used to be mentors to two groups’).

**FIGURE 1:**
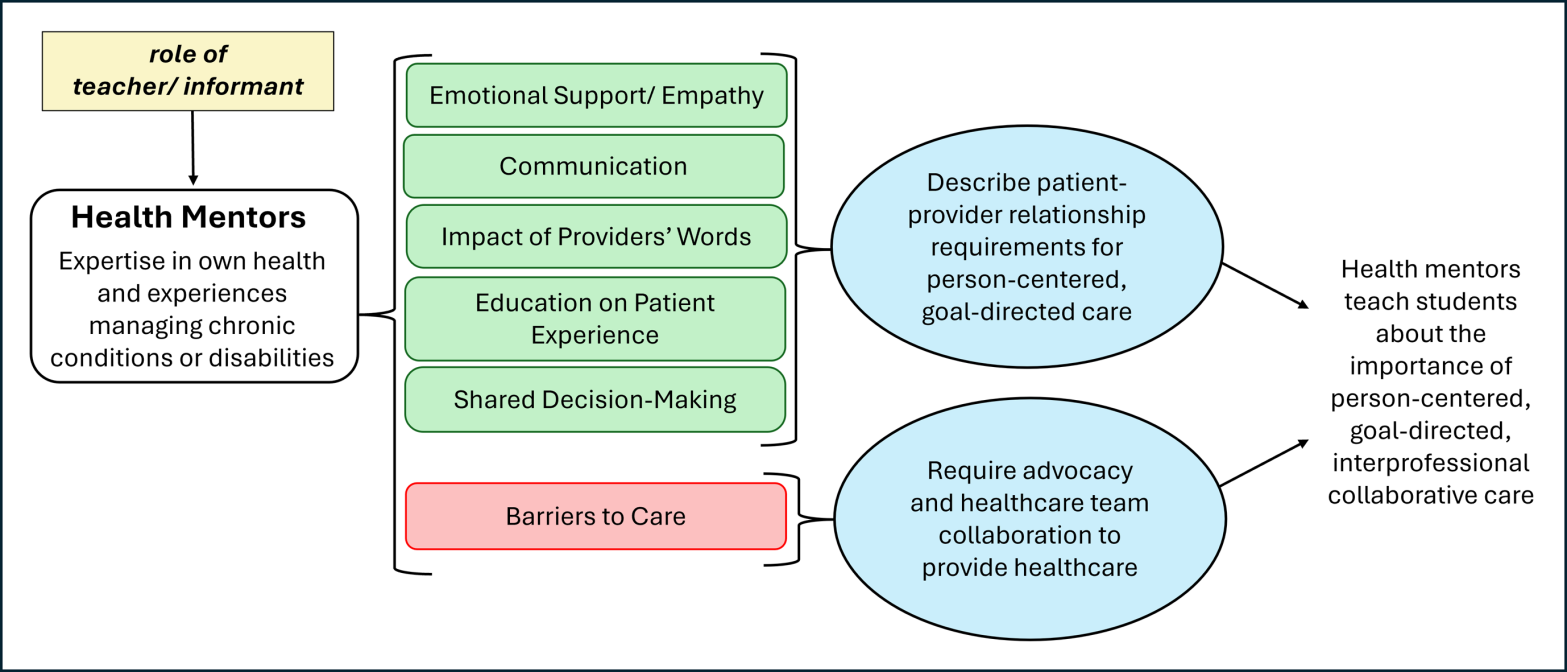
What health mentors share with interprofessional student teams.

The remaining 153 unique thoughts expressed by study participants in focus group sessions described topics pertaining to the study's aims and were assigned distinct codes. The frequency of thought units and exemplary quotations associated with each theme are presented in Tables 3 and 4. Health mentors reported teaching students about a variety of barriers and facilitators to healthcare access and quality during programme meetings. They most commonly described sharing experiences pertaining to environmental barriers followed by insurance, then costs and finally time. Health mentors also reported sharing facilitators associated with each of these themes but to a much lesser extent. Health mentors also taught students about specific experiences with chronic illness, most frequently mentioning thoughts within the themes of personal patient experience and communication followed by person‐centred care, healthcare team collaboration and advocacy. Less frequently mentioned themes included emotional support and impact of providers' words. As expected, almost all themes reflected competencies for interprofessional collaborative practice and disability clinical care, and facilitators and barriers to healthcare previously reported in the literature. One theme, ‘impact of the provider's words’, was not found in our a priori derived codes. A schematic overview of the present studies' findings is presented in Figure 1.

**TABLE 3:** Barriers to healthcare access that Health Mentors share with student teams.

Theme	*N*	%	Exemplary quotation (*source*)
Environmental	18	11.80%	‘If you are if you are [amputated] above the knee. You can walk within the house, but you cannot go down the steps and so you can call a taxi, but the taxi driver cannot get you into the car, so you literally have to take an ambulance’. (pJL_t21)
Insurance	16	10.50%	‘Insurance. They pay for this but they will not pay for that. Like you know what I mean? Like, that's the biggest issue that I'm having or have had for the past 18 years’. (pCC_t19)
Costs	15	9.80%	‘Well, only thing I tell the students is when it comes to hearing aids, $4000. Nobody give me that. I gotta make sure I got something in escrow … So I always make sure in my annual budget I've got whatever I need to get the mould, the hearing aid. You know, financially prepared for that’. (pBB_t13)
Time	13	6.60%	‘I asked for an appointment and they are like, oh, well, he has an appointment in four months. I'm like, no’. (pCW_t13)

*Note: N* = number of thought units. Percentages out of 153 units of thought that met codebook definitions across five focus group transcripts. Sources in parentheses indicate the participant and transcript identifiers for each exemplary quotation.

**TABLE 4:** Experiences with chronic conditions and healthcare that Health Mentors share with student teams.

Theme *(link to competency frameworks)*	*N*	%	Exemplary quotation (*source*)
**The patient experience with chronic conditions, impairments or disabilities**	18	10.80%	‘I asked them do you know or have experiences or anyone in your life with a traumatic brain injury? Chances are most likely with all the students I speak to, they have had no one that they have known or very minimal experience. I take them through what it had done to me’. (pJR_t13)
**Communication** *(IPC C1‐C7; DC, 2.3, 3.3, 4.4, 7)*	20	12.30%	‘Sometimes I have to come with a family or a staff to an appointment with a special needs person. You [the healthcare professional] want to talk to the staff, but why? You cannot talk to him [the patient]? I give them scenarios on like, because, just because, like, I'm myself, I'm hard of hearing. I wear a hearing aid that's why I kind of missed out [on communications]’. (pBB_t13)
			‘My mother used to take the blue pill and the pink pill and a yellow pill, and when you would ask her, ‘What are they for?’ Her answer was ‘The doctor sent them to me.’ She had no clue why she was taking this, and this is something that I've shared with my my the groups throughout the years‐ that not everybody that they will ever encounter, regardless of what their [the students'] profession ends up being, is going to be knowledgeable about what their medication is, when was the last time they had tests done, or what are their symptoms’. (pAM_t12)
**Person‐centred care** *(IPC guiding assumptions; DC 2.1–2.9)*	13	6.60%	‘I talked to the students a lot about building rapport. That you are talking to a person that, they might also be a patient, but talk to the person. And I talked a lot about trust. There's very few docs that I've steered away from at [health center name] but where there's not the feeling of rapport and trust, I find someone else. But it's important that it goes to credibility. You're trying there to treat the whole patient right’. (pMB_t09)
**Healthcare team collaboration** *(IPC, DC 4)*	13	6.60%	‘I express how important it is for communication between the nurse and the doctor and the physical therapist, and all people involved in the program, so that the patient gets the best care’. (pBT_t13)
**Advocacy** *(IPC VE2, DC 4.5, 5 and 8)*	13	6.60%	‘…And they are like this person needs help immediately. Get them on a waiver. Get them the service they need. And then. That just opens the door to getting the help that they that they need in my specific scenario, that's exactly what happened. They went back to like, OK, we need the reports from. The ones that were diagnosable, they went back to like [NAME] Hospital when I was a kid. And then they went forward and they were, and the doctor was like, alright, we have all the ones that are diagnosable and submitted it to the state got on the waiver, got all the help I need it’. (pJR_t13)
**Emotional support/empathy** *(IPC VE7; DC 2 and 7)*	7	3.60%	‘I was going to say one of the biggest things that I try to impress upon them is, well, two things. One is that they are going to be experiencing people at some of the worst moments of their lives. And two, to not begrudge us our worst moments’. (pDP_t09)
**Impact of providers' words on patients**	7	3.60%	‘One piece of advice to my group. I told them that if you are a doctor, you should not whisper about your patient to your physicians assistant. That happened to me one time in the emergency room. That was a big no no’. (pMP_t12)
			‘When I sustained my spinal cord injury. I was told that I would get back whatever I was going to get back within two years. And that two year anniversary was really horrible. Because I thought that this was it. So I always let my groups know that too, that being realistic is different from like dashing dreams. And I think if as providers they can leave the door open even if it's cracked it, it goes really far in the hopefulness’. (pDP_t09)
**Shared decision‐making** *(IPC TT3 and TT9; DC 4.4)*	6	3.10%	‘I thought the doctors here were terrific. All the options that they showed me told me, and actually even inquired to go outside of [name of institution] for another opinion. So I was taken care of very well, so I’. (pRL_t13)

*Note: N* = number of thought units. Percentages out of 153 units of thought that met codebook definitions across five focus group transcripts. Sources in parentheses indicate the participant and transcript identifiers for each exemplary quotation. Under each theme, we indicate the link to the competency frameworks that guided a priori codes in parentheses. IPC = core competency in interprofessional collaborative practice (IPEC, 2023). DC = core competency in disability (NCD, 2022).

## 4 Discussion

Several studies have examined the impact that longitudinal community‐based IPE programmes have on healthcare students and revealed that health mentors, community members with chronic illness who serve as educators, enjoy teaching health professions students. Partial programme theories propose that the mechanisms by which learners develop person‐centredness in these programmes are supported by patients taking on the roles of teachers and informants [17]. Therefore, the present study explored if the conditions of a long‐standing HMP in the United States resulted in expected teaching outcomes. This study is among the first to explore what health mentors teach healthcare students about their experiences as patients with chronic illness, and how these compare to experiences reported by other patients in clinical literature. Drawing on five focus group interviews with health mentors, the experiences shared offer valuable insight into the diverse categories of learning that experiential community‐based IPE curricula provide to future healthcare professionals and highlight possibilities for enhancing care for patients with chronic conditions and disabilities.

By employing focus group interviews, this study offers a solution to the logistical challenges of an observational research approach in capturing the patient voices of health mentors during community‐based IPE programmes. These types of programmes are typically structured to involve multiple meetings and home visits with various student teams, often occurring simultaneously, making it difficult to solicit comprehensive and detailed feedback from community members [7]. Thus, focus group methods allowed for another approach to understand and catalogue health mentor contributions to student learning in these curricula.

This study demonstrates that health mentors share numerous barriers and facilitators to receiving care for their chronic conditions with their student teams that elaborate on previous clinical literature. Patients with chronic conditions outside of community‐based IPE settings have identified a desire for healthcare providers to better understand the environmental challenges that hinder access to care [27]. Consistent with the findings of previous studies, health mentors educated health professions students about barriers such as the cost of care, environmental accessibility and transportation (see Table 3). In addition to these findings, health mentors highlighted the influence of insurance coverage and various time constraints on their ability to obtain adequate healthcare.

*Health mentors share numerous barriers and facilitators to receiving care for their chronic conditions with their student teams*.

Health mentors also reported sharing a variety of personal experiences related to living with and receiving care for chronic conditions (see Table 4), both positive and negative aspects. Information shared by health mentors in the present study reinforces previous findings that patients value when healthcare professionals understand their own unique lived experiences with chronic conditions or disabilities and the impact of person‐centred care [9, 21]. Focus groups with health mentors expanded on this literature, suggesting that individuals with chronic conditions also teach healthcare students about the value of collaborative practice within healthcare teams and the importance of provider advocacy on behalf of patients. Health mentors also highlighted how emotional support is an essential component of their experiences of receiving care for their chronic illnesses by sharing the importance of communication, empathy and the impact providers' words can have on a patient. This study highlights the importance of engaging patients as educators within healthcare student training to impart lessons regarding person‐centred care to future healthcare professionals, as described in prior work [8, 17].

*Individuals with chronic conditions also teach healthcare students about the value of collaborative practice within healthcare teams and the importance of provider advocacy*.

### 4.1 Implications for the Clinical Teacher

Based on prior evaluations of student essays, it is known that students in HMP gain lessons surrounding person‐centred, team‐based, collaborative care when they are asked to reflect [11]. However, it was not known what the health mentors specifically taught students during team meetings. The present study provides insight into the diversity of topics that health mentors have expertise to teach students about. We found that health mentors reported teaching students about every topical area defined in our codebook, which was designed to reflect interprofessional collaboration and disability care competencies. This finding strengthens the case for clinical educators to trust that patients are teaching the importance of person‐centred, goal‐directed, interprofessional collaborative care in student‐led meetings. Paired with previously published findings on student take‐aways, we see an alignment among health mentor lessons and student‐reported learning, which suggests that students have been receptive to these lessons.

Another important implication of the present study of relevance for clinical educators pertains to the question of how to recruit and prepare health mentors to teach lessons aligned with the learning objectives of community‐based experiential IPE programmes like HMP. This study's results align with role theory, which proposes that assigning someone to specific roles can influence their behaviour (e.g., [18]). Given that community members with chronic conditions are already experts in their own health and lives, we found that the orientation of participants to roles during recruitment and training was sufficient to empower patients to take on the role of teachers and informants in HMP. Pragmatically, this offers a benefit for clinical educators who hope to develop, scale and sustain these initiatives that detailed lesson plans are not necessarily needed for successful implementation in this context.

*Orientation of participants to roles during recruitment and training was sufficient to empower patients to take on the role of teachers and informants in HMP*.

### 4.2 Future Directions

Future studies are planned by the research team to analyse the remaining data collected in focus group interviews. These data include events that led the community members to become health mentors, perceptions of their role in the community‐based IPE programme, qualities of effective and ineffective healthcare teams and any observed changes in student team performance throughout the HMP. Understanding this data can allow us to continue to gain direct feedback from community volunteers with chronic illness about their experiences participating in community‐based IPE programmes, and how to best incorporate their feedback within curricula. Future studies can also investigate if specific demographic variables, such as identifying as disabled or geographical location, influence what community members share with their healthcare students.

### 4.3 Limitations

A standard limitation of focus group research is the phenomenon of ‘groupthink’ wherein data may reflect conforming ideas among participants; however, co‐moderators employed recommended strategies to minimize the presence of groupthink during focus group sessions in the present study [28]. In addition, the results presented here represent a purposive sample of health mentors and therefore may not be fully inclusive of the wide variety of experiences from the broader chronically ill population. Specific barriers and facilitators may be more or less emphasized in this population than in other locations and hospitals. In particular, 42% of the participants in the study sample were referred to the health mentors' programme by their provider or another healthcare professional, which may reflect that the participants in our sample have stronger or more positive connections with the healthcare system than chronically ill individuals who do not volunteer as health mentors.

The focus group design also limited our ability to investigate if lessons taught by health mentors with different demographic characteristics were differentially represented among the thematic categories. Focus groups aim to generate a diversity of perspectives, and therefore, moderators or participants may shift discussion to a new topic based on the course of conversation. Some focus group participants may decide not to repeatedly mention a topic if it has been shared by many others already. Therefore, it may be true that all members of the focus group taught students lessons within a specific theme, but only one or two participants had the opportunity to detail it explicitly during the session.

## 5 Conclusions

Focus group interviews with health mentors who volunteered to teach healthcare students through community‐based IPE highlighted the importance of incorporating experiences of patients with chronic conditions and disabilities within healthcare curricula and orienting all participants in HMP to patients in the role of teachers. Health professions educators can rely on patients with chronic illness to teach future healthcare professionals using their unique stories so students can better understand facilitators and barriers to care, a variety of chronic conditions and disabilities and components that positively and negatively impact healthcare quality in semi‐structured student‐led contexts.

*Health professions educators can rely on patients with chronic illness to teach future healthcare professionals using their unique stories*.

## Author Contributions

**Rachel White:** conceptualization, data curation, formal analysis, funding acquisition, investigation, methodology, project administration, resources, visualization, writing – original draft preparation. **Maria Brucato:** conceptualization, data curation, formal analysis, investigation, methodology, project administration, resources, software, supervision, visualization, writing – original draft preparation, writing – review and editing. **Amber King:** formal analysis, funding acquisition, writing – review and editing. **Anne B. Mitchell:** formal analysis, supervision, writing – review and editing. **Nethra S. Ankam:** conceptualization, supervision, writing – review and editing.

## Ethics Statement

The research protocol was approved by Thomas Jefferson University's Institutional Review Board (RB#21E.510).

## Consent

Verbal informed consent was obtained from all study participants.

## Conflicts of Interest

The authors declare no conflicts of interest.

## Supporting information


**Appendix S1:** Supporting information.

**Appendix S2:** Supporting information.

**Appendix S3:** Supporting information.

## Data Availability

To protect participant privacy, focus group transcript data are not openly available. Inquiries regarding data can be directed to the corresponding author.
