# Supplementary material for: What Community Members With Chronic Illness Teach Future Healthcare Professionals in a Longitudinal Interprofessional Education Program: A Focus Group Study
Source: Clin Teach. 2025 Aug 15;22(5):e70181. doi: 10.1111/tct.70181 (PMC12357013; doi:10.1111/tct.70181)
Supplement: Supplementary file 1 — Appendix S1: Supporting information. [file TCT-22-e70181-s002.docx]

Appendix 1: Demographics Survey

Jefferson Heath Mentors Program

Focus Group Demographics

Q1 Please enter your name below.

- First Name _____________________________________
- Last Name _____________________________________

Q2 Please select the range that includes your age:

- 18-24
- 25-34
- 35-44
- 45-54
- 55-64
- 65-74
- 75-84
- 85-94
- 95+

Q3 What is your zip code?

______________________________________________________

Q4 What is your sex assigned at birth?

- Male
- Female
- Prefer not to say

Q5 Which racial or ethnic groups best describe you?

*Please select all that apply.*

- African American/Black American
- Asian or Pacific Islander
- Caucasian/White
- Hispanic/Latino
- Indian or Alaskan Native
- Multi-racial
- Other, please specify _____________________________
- Prefer not to say

Q6 Do you volunteer in Health Mentors to share your experience as:

- A patient
- A caregiver
- Both

Q7 How were you referred to the Health Mentors Program?

- From a current health mentor
- From my provider
- From a recruitment session
- From Jefferson Center for Interprofessional Practice and Education (JCIPE) website
- From a posted flyer
- Other, please specify:__________________________________

Q8 How many years have you volunteered in the Health Mentors Program?

_____________________________________________________

Q9 Do you participate in any other volunteer activities outside of Health Mentors Program?

- Yes (please briefly explain below)

______________________________________________________

- No

Q10 Please list chronic condition(s)/impairment(s)

________________________________________________________

________________________________________________________

_________________________________________________________

_________________________________________________________

_________________________________________________________

_________________________________________________________

_________________________________________________________

Q11 How many years were you diagnosed with at least one chronic condition/impairment before becoming a Health Mentor volunteer

- Less than 1 year
- 1-5 years
- 6-10 years
- 10+ years

Q12 How disabling are your current condition(s) to your daily functioning?

- No impact
- Slight Impact
- Moderate Impact
- Fair Impact
- Significant Impact

Q13 Do you identify as having a disability?

- Yes
- No
- Unsure
- Prefer not to say

**Please answer the following questions only if you participate in the Health Mentors program to share your perspectives as a caregiver, or BOTH a patient and caregiver.**

*(If you answered “A caregiver” or “Both” to Q6)*

Q14 What is your relationship to the person that you care for?

______________________________________________________

Q15 How many years were you their caregiver before becoming a Health Mentor volunteer?

- Less than 1 year
- 1-5 years
- 6-10 years
- 10+ years

Please answer the following questions about the individual that you care for. If you care for more than one individual, please choose one person to answer the following questions about.

Q17 Please select the range that includes their age:

- 18-24 (1)
- 25-34 (2)
- 35-44 (3)
- 45-54 (4)
- 55-64 (5)
- 65-74 (6)
- 75-84 (7)
- 85-94 (8)
- 95+ (9)

Q18 What is their zip code?

___________________________________________________

Q19 What is their gender identity?

- Male
- Female
- Non-binary
- Prefer not to say

Q20 Which racial or ethnic groups best describe them?

*Please select all that apply.*

- African American/Black American
- Asian or Pacific Islander
- Caucasian/White
- Hispanic/Latino
- Indian or Alaskan Native
- Multi-racial
- Other, please specify ______________________________
- Prefer not to say
- Unsure

Q21 Please list any of their chronic condition(s)/impairment(s)

________________________________________________________

_________________________________________________________

_________________________________________________________

_________________________________________________________

_________________________________________________________

Q22 How disabling are their current condition(s) to their daily functioning?

- No impact
- Slight Impact
- Moderate Impact
- Fair Impact
- Significant Impact
- Unsure

Q23 Do they identify as having a disability?

- Yes
- No
- Unsure
- Prefer not to say
