# Supplementary material for: What Community Members With Chronic Illness Teach Future Healthcare Professionals in a Longitudinal Interprofessional Education Program: A Focus Group Study
Source: Clin Teach. 2025 Aug 15;22(5):e70181. doi: 10.1111/tct.70181 (PMC12357013; doi:10.1111/tct.70181)
Supplement: Supplementary file 2 — Appendix S2: Supporting information. [file TCT-22-e70181-s003.docx]

**Appendix 2: Focus Group Guide**

**Focus Group Discussion Guide**

**Introduction of Research Team**

Hello, my name is [Name of moderator], I use [pronouns] pronouns, and I’ll be your moderator today. I will be leading today’s discussion. My role is to ask you all questions, keep to the timeframe, and make sure that you all have the chance to share your knowledge and experiences.

This is [Name of note-taker], and they will be taking notes during the session today. Thank you for agreeing to participate in this focus group about your experience in the Jefferson Health Mentors Program!

***If OHR-8H consent process occurred in a previous study session, you must repeat verbatim the script from OHR-8H to confirm the participant is still willing to participate given the study details.**

**Ground Rules:**

Let me begin our discussion by reviewing a few things about the focus group.

We will be focusing on some specific topics. We are interested in what everyone has to say about them. If someone shares an idea that you want to expand on, or if you have a different point of view, please speak up.

Sometimes I may have to interrupt the discussion to bring us back to the topic or to move on to another question or topic, to make sure that we cover everything on our agenda.

As we move through the agenda today, we will follow some practical guidelines:

- First, we want everyone to express their opinions about the discussion topics. We are interested in different points of view. There are no right or wrong answers.
- **Today, we want to focus on *understanding* your views** rather than finding solutions or agreements about any potential challenges.
- We will ask you to only use first names or nicknames in our discussion today. We hope you will help protect others’ privacy by not discussing details outside the group.
- Please do not hold side conversations during the session. We want to be able to hear from everyone, and to be able to hear what everyone says.
- Sometimes we will go around the table to share views on a topic. You can always “pass” if you prefer not to comment on that particular topic.
- Because we are also audio-recording the session, it would really help us if you could speak up.

Do you have any questions so far?

**Ice-Breaker**

Now, let’s go around the room and have each of you introduce yourselves; give your first name or nickname, your pronouns if you feel comfortable sharing with the group, and how long you’ve been a volunteer in the Health Mentor’s program.

Now, let’s talk more about your experience with the Health Mentor’s Program.

**Prompt 1: Journey to the Health Mentors Program**

First, I’d like you to think back to when you first considered volunteering for the Health Mentors Program.

Can you walk me through the events that led you to volunteer for the Program?

- How was your relationship with your healthcare providers at that time?
- What were your main motivations for volunteering?

**Prompt 2: Health Mentors Program**

Now, I want to explore what exactly the Health Mentors Program means to you.

How would you describe the Health Mentors Program to someone who is unfamiliar to it?

- What is the purpose of the program?
- What is your role in the program?

Next, I want you to reflect on how supported you feel during your time as health mentor.

- What factors contributed to how supported you felt when you were starting as a mentor?

**Prompt 3: Teaching about your Lived Experience**

Thanks for sharing all that with me. Now that we’ve discussed a little bit about who you are, I would love to hear about what you share when interacting with the students within your health mentors group.

**A. What did you hope to teach the students about your lived experience?**

**B. Some patients with chronic illnesses say they experiences things that make it more difficult for them to get the healthcare they need. Have you experienced anything that made it difficult for you to get the care you needed?**

- Did you share this with your team?
- On the other hand, is there anything you’ve found makes it easier for you to receive the healthcare you need?

**C. Next, I would like you to picture an effective healthcare team that meets all of your needs.**

- What qualities do they have?
- What do they do well?
- How do they communicate with you?
- What do they do that makes you feel cared for?
- How do you impart that information to the student teams you mentor?

**D. On the contrary, I now want you to reflect on negative experiences with ineffective healthcare teams.**

- What made them ineffective?
- What was that experience like?
- How do you impart that information to the student teams you mentor?

**Prompt 4: Observation of Student Outcomes**

Thank you for sharing this information about what you teach your students as a Health Mentor. Lastly, I want to explore how teaching the students makes you feel.

I now want you to imagine an effective health mentor at teaching students about their lived experience, here that be yourself or one of your peers you have observed.

- How receptive were students to your patient experience?
- Did you notice any improvements in your students around their person-centered care, or care that is informed by your individual goals and preferences?
- Teamwork?
- If so, how could you tell they improved?

That covers all the topics I wanted to ask you about. **Is there anything we haven’t covered that you think is important to share about your experience in the Health Mentors Program?**

Thank you very much for participating in this focus group. The information you have provided has been very helpful. It will be used to help the Health Mentors Program make informed decisions about ways to improve the program and health professions students’ education.

Are there any questions that I can answer before we end the session?

Thank you again for your help. We really, really appreciate your time and your knowledge.
