# Supplementary material for: What Community Members With Chronic Illness Teach Future Healthcare Professionals in a Longitudinal Interprofessional Education Program: A Focus Group Study
Source: Clin Teach. 2025 Aug 15;22(5):e70181. doi: 10.1111/tct.70181 (PMC12357013; doi:10.1111/tct.70181)
Supplement: Supplementary file 3 — Appendix S3: Supporting information. [file TCT-22-e70181-s001.docx]

**Appendix 3: Codebook**

| **Code** | **Definition** |
| --- | --- |
| Person-Centered Care | HM mentions inclusion or lack of the following in their care:  Providers...  -Demonstrated person-centered care in terms of building a trusting partnership between patients and healthcare providers.  -Devoted time to asking patients about their life outside of their disease. Building rapport.  -Incorporated patient's lifestyle and opinions when choosing treatment plan. |
| Shared decision making | HM mentions inclusion or lack of the following in their care:  Providers…  - Consulted patients before interaction with medical devices (e.g., wheelchair, crutches, service animal, etc.).  - Chose a treatment plan together with the patient.  - Demonstrated skill in engaging the patient and caregivers in the supported decision-making process. |
| Provider Collaboration | HM describes any of following:  - Team approaches to supporting people in health care systems (e.g., interdisciplinary, multidisciplinary, interprofessional) including team reasoning, problem-solving, and decision making among providers/ team members.  -Impact of teams and the specific responsibilities of team members from different professions in addressing health needs and in partnering with patient as a central member of the team.  - Systems of community-based services and supports that may be useful for patients outside of the clinical care system. *  - Communication between healthcare team members (with each other, not with patient) |
| Communication | HM mentions either inclusion or lack of the following in their care:  Providers…  - Understand the patient should be the primary source of information regarding their care.  - Communicate one's roles and responsibility clearly.  - Communicate clearly with authenticity and cultural humility, avoiding discipline-specific terminology.  - Examine one's position, power, role, unique experience, expertise, and culture towards improving communication and managing conflicts.  - Contact them about lab or imaging results within proper time.  - Responses on portal from healthcare team. |
| Emotional Support/ Empathy | HM mentions either inclusion or lack of the following in their care:  Providers…  - Recognize that mental health conditions can be the primary disability condition.  - Recognize the risk of misdiagnosing mental health concerns in patients with disabilities.  - Displays empathy.  - Asks about patient's emotional responses to health conditions.  - Ask about social support at home. |
| Advocacy | HM mentions either inclusion or lack of the following in their care:  Providers…  - Use strategies or supports to accommodate patients with functional limitations (mobility, sensory, cognitive, behavioral).*  - Advocate for unique needs to other healthcare professionals, the patient's workplace, or other. |
| Cost | HM mentions the cost of:  - Medications.  - Copays, visits, or procedures .  - Traveling to health appointments.  - Necessary medical devices or mobility aids. |
| Insurance | HM mentions insurance having an impact on the care/ treatments they receive |
| Time | HM mentions:  - Wait times to see specialists.  - Time spent managing healthcare.  - Time spent on the phone with insurance or pharmacy.  - Time spent traveling to proper healthcare. |
| Environmental Barriers | HM mentions:  - Transportation regarding accessing healthcare.  - Finding childcare coverage during their healthcare appointments.  - Missing time from work.  - Barriers to getting to any healthcare appointment due to physical health.  - Limitations due to lack of accessibility. |
| Education about the Patient Experience | HM mentions:  - teaching students about specific condition, what it feels like to be a patient. |
| Words Impact on Patients | Impact a provider's words have on patients during a vulnerable moment in their life. |
| Other | Does not fit any of the other codes in the codebook |
